# Bladder cancer cell growth and motility implicate cannabinoid 2 receptor-mediated modifications of sphingolipids metabolism

**DOI:** 10.1038/srep42157

**Published:** 2017-02-13

**Authors:** Arianna Bettiga, Massimo Aureli, Giorgia Colciago, Valentina Murdica, Marco Moschini, Roberta Lucianò, Daniel Canals, Yusuf Hannun, Petter Hedlund, Giovanni Lavorgna, Renzo Colombo, Rosaria Bassi, Maura Samarani, Francesco Montorsi, Andrea Salonia, Fabio Benigni

**Affiliations:** 1 Division of Experimental Oncology/Unit of Urology; URI; IRCCS Ospedale San Raffaele, Milan, Italy; 2 Department of Medical Biotechnology and Translational Medicine, University of Milano, Segrate, Italy; 3 Department of Pathology, IRCCS Ospedale San Raffaele, Milan, Italy; 4 Department of Medicine, University of Stony Brook, Stony Brook, New York 11794, USA; 5 Department of Clinical Pharmacology, Linköping University, Sweden; 6 Università Vita-Salute San Raffaele, Milan, Italy

## Abstract

The inhibitory effects demonstrated by activation of cannabinoid receptors (CB) on cancer proliferation and migration may also play critical roles in controlling bladder cancer (BC). CB expression on human normal and BC specimens was tested by immunohistochemistry. Human BC cells RT4 and RT112 were challenged with CB agonists and assessed for proliferation, apoptosis, and motility. Cellular sphingolipids (SL) constitution and metabolism were evaluated after metabolic labelling. CB1-2 were detected in BC specimens, but only CB2 was more expressed in the tumour. Both cell lines expressed similar CB2. Exposure to CB2 agonists inhibited BC growth, down-modulated Akt, induced caspase 3-activation and modified SL metabolism. Baseline SL analysis in cell lines showed differences linked to unique migratory behaviours and cytoskeletal re-arrangements. CB2 activation changed the SL composition of more aggressive RT112 cells by reducing (p < 0.01) Gb3 ganglioside (−50 ± 3%) and sphingosine 1-phosphate (S1P, −40 ± 4%), which ended up to reduction in cell motility (−46 ± 5%) with inhibition of p-SRC. CB2-selective antagonists, gene silencing and an inhibitor of SL biosynthesis partially prevented CB2 agonist-induced effects on cell viability and motility. CB2 activation led to ceramide-mediated BC cell apoptosis independently of SL constitutive composition, which instead was modulated by CB2 agonists to reduce cell motility.

Bladder cancer (BC) is the most common malignancy form of the urinary tract. Identification of novel potential targets to reduce recurrence and prevent disease progression still represents a medical need for this pathology. A variety of putative novel biomarkers or targets have been recently described for urothelial carcinoma^1^. In this context, the endocannabinoid system (ECS) expressed by the genitourinary organs has recently gained particular attention^2^.

Both clinical and experimental evidence suggested a possible role of the ECS in modulating cancer proliferation, progression, and metastasis^3^ in several types of neoplastic diseases, including prostate and breast cancer^4^,^5^. However, little information is currently available on the role of ECS components in proliferation^6^ and metastasis of human urothelial carcinoma.

Cellular SL are components of the cell plasma membranes that change dramatically during differentiation and malignant transformation^7^. Aggressive, muscle-invasive BC is in fact characterized by a different expression of the GM3 and Gb3 glycolipids^8^,^9^, suggesting that the metabolism of SL may control cell invasion and motility^10^.

The known antiproliferative activity of ECS modulators (i.e. cannabinoids) in various cancer models is reported to modify SL metabolism, which typically results in accumulation of ceramide (Cer) via de novo synthesis^11^,^12^ and deregulation of the Cer/ S1P rheostat^13^.

Thus, the effects CB2-receptor activators on BC survival and motility and their interactions with SL biochemical pathways represent an interesting aspect still poorly characterized.

The present study investigates the expression and activity of CB2 receptor in human BC cells and its interaction with plasma membrane SL, which may modulate BC survival and progression. As a result, the association between some ECS components, such as CB2, and SL metabolism in bladder malignancy, may in turn represent an interesting therapeutic option to be further investigated.

## Results

### Human bladder cancer cells upregulate CB2 receptor

**Figure 1:**
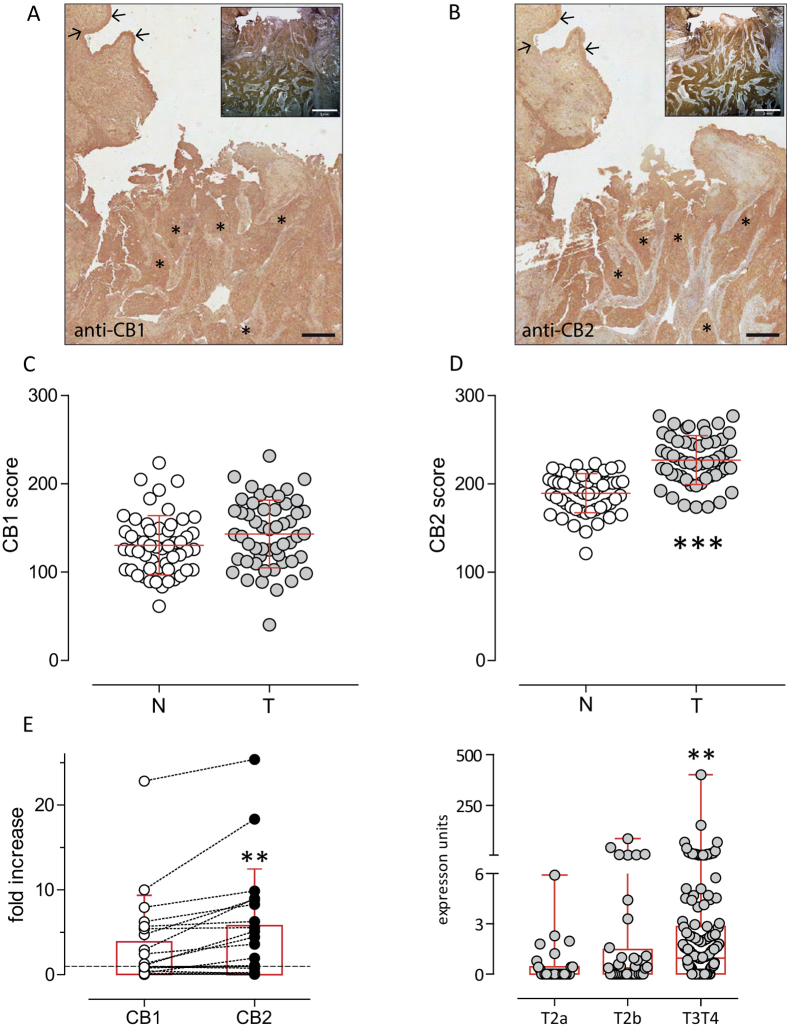
Expression of CB in human BCa. Representative immunoreactivity (black scale bars: 500 μm) of cannabinoid CB1 (panel A) and CB2 (panel B) receptors from a representative patient with muscle invasive bladder carcinoma. Image insets are lower magnification of the representative sample to evidence muscle-infiltrating tumor specimen (white scale bars: 2 mm). Tumour lesion (asterisk) and normal urothelium (arrows) are indicated. Formalin-fixed paraffin-embedded sections from 53 different tumour specimens stained with haematoxylin and CB1 and CB2 antibodies (DAB) were analyzed with the Color Deconvolution analysis tool to quantify the DAB staining intensity. Staining score of CB1 (panel C) and CB2 (panel D) receptors were measured on the tumour lesion (T, grey symbols) and normal epithelium (N, white symbols) of 53 different patients in duplicate slides. Data are shown individual values. The mean ± sd is shown. ***p < 0.001 vs normal tissue, Paired Student t-Test. Control staining in the presence of displacing CB1 and CB2 peptides are shown in Suppl. Fig. 1. Panel E: CB1 and CB2 gene expression analysis of primary muscle invasive BC frozen specimens (N = 19) compared to paired healthy tissue (dashed line) by quantitative PCR analysis. Data are represented as fold-increase values depicted both as individual values (mean of quadruplicates) of matching CB1-CB2 expression and as mean ± sd. **p < 0.01 vs CB1, Paired Student’s T test. Panel F: in silico analysis of the expression of CB2 receptors in primary muscle-invasive bladder cancers. Data are expressed as individual values and box-and-whiskers plots encompassing 28 samples of T2a tumours, 37 samples of T2b tumours, and 137 samples of T3T4 tumours. Error bars represent the max value in each group. **p = 0.0015, 1-way Kruskal-Wallis ANOVA with Dunn’s multiple comparison post test.

Immuno-histochemistry showed specific reactivity for both CB1 and CB2. In 53 normal-tumour paired specimens, signal for CB1 appeared similar in healthy and neoplastic areas (Fig. 1A–C), while signal for CB2 increased in tumour tissue (Fig. 1B–D). The immunoreactivity of both CB1 and CB2 was specific, as confirmed by control staining with competing peptides (Suppl. Fig. S1). Quantitative PCR analysis in frozen specimens (n = 19) from primary muscle-invasive bladder tumours, compared to paired normal tissue, showed both CB1 and CB2 mRNA upregulation but the level of CB2 was higher (Fig. 1E). Moreover, the expression of *CB2* mRNA, analysed *in silico* by interrogating the Cancer Genome Atlas dataset available on cBioPortal for Cancer Genomics^14^,^15^ in BC patients, was found significantly higher in advanced tumours (Fig. 1F). Exhaustive genomic and bioinformatics analysis of CB2 in BC is reported in supplemental results. Finally, mRNA and protein expression of CB1 and CB2 was confirmed in RT4 and RT112 cell lines (Suppl. Fig. S2A,B).

### CB2 receptor mostly contributes to the cannabinoid-induced cytotoxicity in bladder cancer cell lines

**Figure 2:**
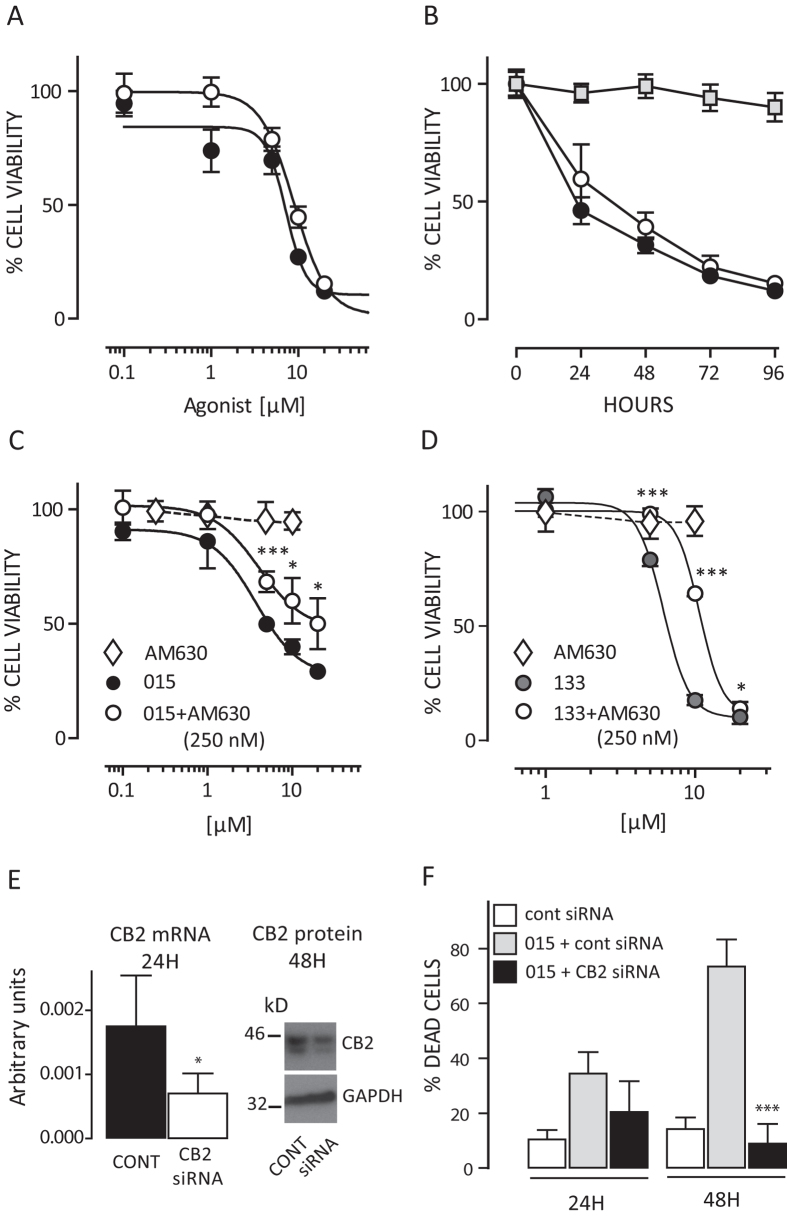
Effect of cannabinoids on bladder cancer cell proliferation. Panel A: Dose-response cytotoxicity (MTT assay) on RT112 (black symbols) and RT4 (white symbols) cells, measured 72 hours post JWH015. Panel B: Kinetics of cytotoxicity after 20 μM JWH015 on both RT4 (white symbols) and RT112 (black symbols) cells. The effect on normal fibroblasts is also shown (grey square symbols). Panels C and D: Effect of the CB2 antagonist AM630 (0.25 μM) on CB-2 agonist-induced RT112 cell toxicity. Cells were pre-treated with AM630 for 30 min before exposure to either different concentrations of JWH015 (015, C) or JWH133 (133, D). Cell viability was assayed (MTT assay) 72 hrs post agonist challenge. Effect of antagonist alone (AM630, 0.25–10 μM range, white diamonds) is shown. *p < 0.05, ***p < 0.001 vs agonist alone, 2-ways ANOVA, Bonferroni post-test. Data are represented as mean ± sd of quintuplicate values. A single experiment is shown as representative of three independent ones with similar results. Panel E: down-modulation of CB2 mRNA (qPCR) and protein (Western blot) in RT112 cells after specific gene silencing. *p < 0.05 vs control siRNA (T test). Panel F: Effect of CB2 genetic silencing 24 and 48 hrs post JWH015 (015, 20 μM) on cytotoxicity. Data are expressed as mean ± SD of quadruplicate cell counts (Trypan Blue exclusion). ***p < 0.001 vs 015+cont siRNA (1-way ANOVA, Dunnett’s post test).

The preferential CB2 agonist JWH015^16^,^17^ induced antiproliferative activity on both RT4 and RT112 cells (Fig. 2A), with a specific IC50 value around 5 μM. Cell viability reduced by 85 ± 5% and 88 ± 8% (*p* < 0.001) in RT4 and RT112 cells respectively, 4 days after agonist exposure (Fig. 2B). Pre-treatment with the CB2 antagonist SR144256 reduced, after 72 hrs, the JWH015-induced (20 μM) cytotoxic effect by 25.6 ± 3% (*p* < 0.01 vs JWH015 alone, not shown). A second, more potent CB2 antagonist (AM630) rescued significantly the RT112 cytotoxicity after both JWH015 and JWH133, a more CB2-selective agonist^18^ (Fig. 2C,D). No significant effects by CB2 antagonists alone (up to 10 μM) were detected. JWH015 induced similar cytotoxicity in T24, HT1376, and 5637 BC cell lines (not shown) but did not affect the viability of human primary fibroblasts (Fig. 2B). Genetic silencing of CB2 receptor in RT112 cells reduced both mRNA and protein (Fig. 2E), inhibited the agonist-induced cytotoxicity (Fig. 2F), and increased cell viability by 42.9 ± 16% after JWH015 (20 μM) challenge (MTT assay, *p* < 0.05 vs control siRNA).

The relative role of CB1 and CB2 in cytotoxicity was tested by using anandamide (AEA, CB1/CB2 dual agonist). Data (Suppl. Fig. S3) support the major contribution of CB2 in controlling BC cell survival. Finally, intravesical instillations of JWH015 (20 μM), started three days after intramural orthotopic RT112-luc implantation (see Supplemental Methods), significantly reduced tumour burden (Suppl. Fig. S4A).

### Cytotoxicity Mechanism of Action by synthetic CB2 agonist in RT112 cells

**Figure 3:**
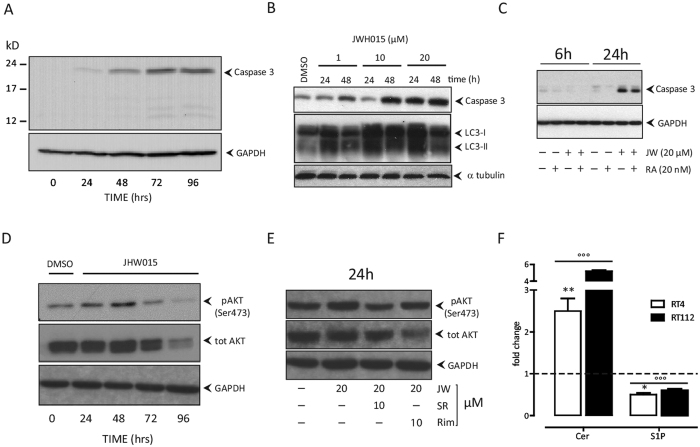
Molecular events driving JWH015 cytotoxicity. Western blot analyses in RT112 cells: Panel A: JWH015-mediated (20 μM) induction of caspase 3 over time (0–96 hrs). Panel B: Dose-effect of JWH015 (1–20 μM) in inducing caspase 3 and LC3I-II 24–48 hrs post exposure. Panel C: JWH015-mediated (JW, 20 μM) induction of caspase 3 6–24 hrs after exposure with or without addition of rapamycin (RA, 20 nM). Panel D: modulation of pAKT by JWH015 (20 μM) over time. Untreated (DMSO) control cells tested both at time 0 and after 96 hrs of culture were similar. Panel E: Effect of CB1 (rimonabant, Rim, 10 μM) or CB2 (SR14452, SR, 10 μM) antagonists on pAKT when simultaneously administered to cells together with the CB2 agonist (JWH015, JW, 20 μM) after 24 hrs incubation. A single experiment is shown as representative of three independent ones with similar results. Panel F: Ceramide (Cer)/sphingosine 1-phosphate (S1P) rheostat levels in RT4 and RT112 cells treated with JWH015 (20 μM). Cell lipids were metabolically labelled with [1-^3^H]sphingosine after 48 hrs JWH015 incubation. Radioactive lipids were visualized by digital autoradiography and quantified with specific Beta-Vision software. Data are expressed as fold-change with respect to untreated control cells (dashed line) and are the mean ± SD of three different experiments. °°°p < 0.001 vs untreated cells baseline (dashed line); *p < 0.05 vs RT112. **p < 0.01 vs RT112, 2-tail unpaired Student’ T test.

*In vitro*, the apoptotic phenotype of cells (annexin-V^+^/PI^+^) increased 6.7 ± 0.3 fold (p < 0.01) 48 hrs post JWH015 (Suppl. Fig. S5A). Treated cells also accumulated in the sub-G1 phase (28.2 ± 1 vs 2.2 ± 0.9% in control cells, *p* < 0.01) (Suppl. Fig. S5B). Apoptosis was confirmed by activated form of caspase 3 that increased following JWH015 (Fig. 3B). Caspase induction was not detectable earlier than 24 hrs after CB2 agonist exposure on RT112 cells, even in the presence of rapamycin (Fig. 3C). After an initial up regulation detected 24–48 hrs post drug exposure, AKT phosphorylation (pAKT) was significantly reduced at day 3 and 4, consistently with the cytotoxic effect (Fig. 3D). The up regulation of pAKT in RT112 cells 24 hrs post JWH015 was reversed by co-treatment with CB2 antagonist (SR144528) but not with CB1 antagonist (rimonabant) (Fig. 3E), suggesting a selective receptor-dependent effect.

In RT112 cells, JWH015 induced early autophagy with LC3 conversion (Fig. 3B; Suppl. Fig. S6A–C) and reduced phosphorylation of p70S6 (mTORC1 inactivation)(Suppl. Fig. S6D).

### CB2 activation in BC cells induces changes in SL metabolism

Independently of their different SL basal composition (Suppl. Fig. S7), JWH015-treated RT112 and RT4 cells exhibited 2–5-fold (*p* < 0.001) increased Cer levels over the baseline of untreated cells (Fig. 3F), while the pro-survival signal S1P was reduced by approx. 40% in both cell lines (*p* < 0.001; Fig. 3F). Exposure to JWH015 did not change total sphingomyelin (SM, Suppl. Fig. S7) and sphingomyelinase (SMase, Table 1) activity, while activities of plasma membrane glycohydrolases GBA1/GBA2, β-galactosidase, and β-hexosaminidase increased significantly (Table 1). Contribution via *de novo* synthesis to the final Cer level in JWH015-treated cells was confirmed by approx. 2-fold induction of Cer synthase mRNA in RT112 and RT4 cells (not shown).

### CB2 receptor engagement contributes towards control of SL-dependent motility in aggressive BC cells

RT4 and RT112 cell lines expressed different SL (Suppl. Fig. S7) and behaved like low and high aggressive tumour cells respectively (Suppl. Fig. S8).

**Figure 4:**
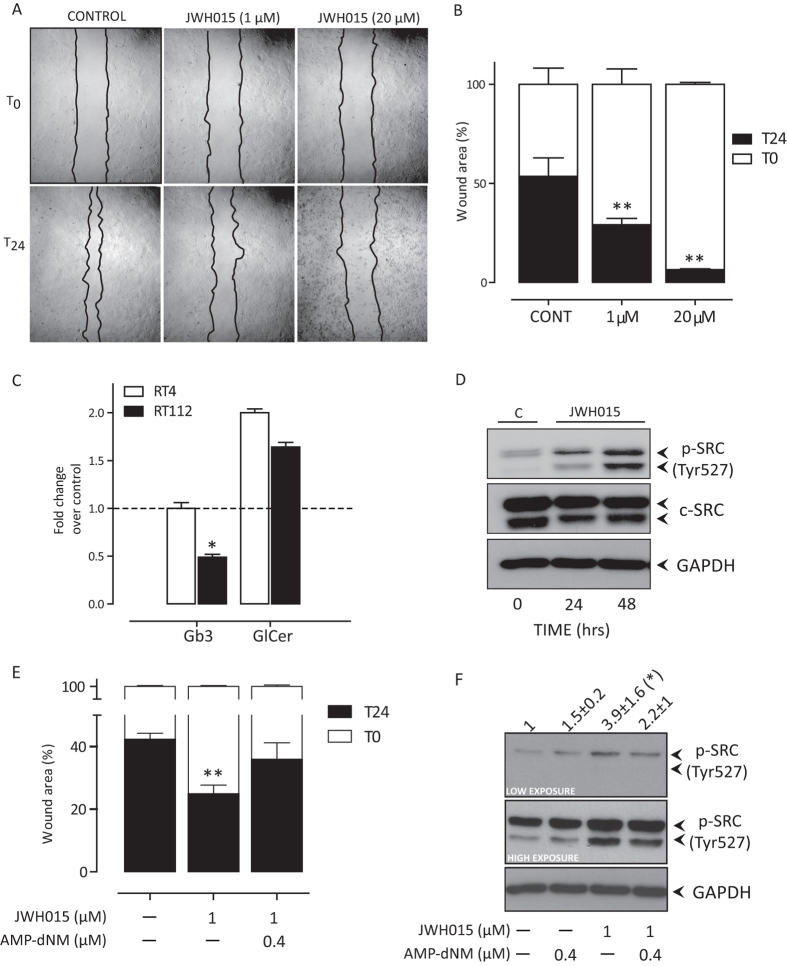
Effect of JWH015 treatment in high-motility RT112 cells. Panels A: Representative images of RT112 wound healing assay evaluated 24 hours post scratch. Wound edges were artificially highlighted by drawing a black line. Panel B: Cell migration was quantified as % of cell-covered area at 24 hours (black bars) compared to the cell-free wound area at time 0 (white bars). Data are the mean ± sd of three independent experiments in triplicate. **p < 0.01 vs T0, 1-way ANOVA, Dunnett’s post test. Panel C: Levels of SL (Gb3, and GlcCer) in RT4 and RT112 cells 24 h after JWH015 (1 μM) exposure. Data are the mean ± sd of three independent experiments and expressed as fold change over the level measured in untreated cells (indicated by the dashed line). *P < 0.05 vs RT4, 1-way ANOVA, Dunnett’s post test. Panel D: immunoblot analysis of inactive c-Src (pSRC Tyr527) 24 and 48 hours after JWH015 (1 μM) exposure in RT112 cells. Similar results were obtained in two other independent experiments. Panel E: Effect of SL synthesis inhibition by AMP-dNM (0.4 μM) on cell migration of RT112 treated with JWH015 (1 μM); data are the % (mean ± sd, n = 4)of re-populated scratched area. Panel K: Immunoblot analysis of inactive c-Src (pSRC Tyr527) in RT112 cells treated with JWH015 (1 μM), with or without AMP-dNM (0.4 μM). The calculated fold-increase of band density (mean ± sd, n = 4) is shown above the panel. Both low and high exposure blots are shown.

In a scratch assay, 1 μM JWH015 significantly inhibited, by approx. 50%, reconstitution of cell monolayer (Fig. 4A,B), 24 hrs after stimulation. Higher concentration of JWH015 (20 μM) reduced cell migration by almost 90% (Fig. 4A,B) but the frequency of apoptotic and necrotic cells increased (40 ± 3%, p < 0.01 vs 3 ± 2% 1 μM JWH015 or vs 3.5 ± 2% untreated cells). The reduced motility in RT112 cells after JWH015 was associated to lower Gb3 levels, similar to those ones measured in low-motility RT4 cells, (Fig. 4C, *p* < 0.01).

Furthermore, the inactive form of c-SRC, which is typically phosphorylated in Tyr-527, was up-regulated over time in RT112 treated with the CB2 agonist (Fig. 4D). Finally, the effect of JWH015 on cell migration was partly reverted by the AMP-dNM-mediated inhibition of SL metabolism (Fig. 4E,F) and CB2 genetic silencing completely prevented the JWH015-mediated increase of inactive pSRC (not shown).

## Discussion

Consistently with previous data^19^,^20^, we found that, in primary BC, the expression of CB2 in the neoplastic lesion is higher when compared to the paired normal urothelial and stromal counterparts. Our results also show that, like in other tumours^21^,^22^,^23^ CB2 receptors may selectively control some cellular functions in BC cells.

From a tumour-evolution point of view, these data may appear in contrast, as CB2 agonists block cancer proliferation and migration. Nevertheless, even though receptor/agonist interactions of ECS components is quite complex and far to be fully elucidated, this receptor up-regulation may actually reflect a tumour strategy to favour survival; in fact in physiological conditions, sub-micromolar concentrations of (endo)cannabinoids are reported to induce either mitogenic signals^24^,^25^ or down-modulate locally the immune system^26^.

Altogether, our data support the specific use of CB2 as antitumor target^27^ also in BC, but we cannot exclude the involvement of other ECS receptors, such as GPR55, which may interact with JWH015^28^,^29^ promoting cell proliferation but also apoptosis^30^,^31^,^32^.

Similarly to other systems^23^,^33^, BC cells react to the cannabinoid-induced stress by activating autophagy as defective survival signal that eventually ends up to apoptosis. In this context, Cer, a pro-apoptotic lipid acting as second messenger of cannabinoids^34^, inhibits of the pro-survival signal Akt via mTORC1 inactivation^35^,^36^. We indeed detected a late and significant down-modulation of Akt preceded by a transient increase of pAkt. This initial Akt activation may be explained by either a pro-survival cellular homeostatic loop as previously described^37^, or by a possible effect on the GPR55 receptor that is reported to upregulate Akt in other tumours^30^,^31^.

With respect to SL metabolism, CB2 activation has been shown to induce cell apoptosis through the stimulation of *de novo* synthesis of Cer in a number of human tumours, including glioma^12^,^38^, leukemia^39^, and pancreatic cancer^40^. We showed that CB2 activation stimulated Cer synthesis in BC cells thru both conversion of S1P^41^ and de *novo* biosynthesis. This mechanism reflects involvement of the sphingolipid rheostat (i.e. S1P/Cer ratio) that controls cell proliferation and apoptosis^13^ as well as activation of different membrane-associated SL-converting glycohydrolases^42^. Our data also suggest an impact of CB2 activation in BC cell motility and aggressiveness via the inactivation of the FAK-Src pathway^43^.

**Figure 5:**
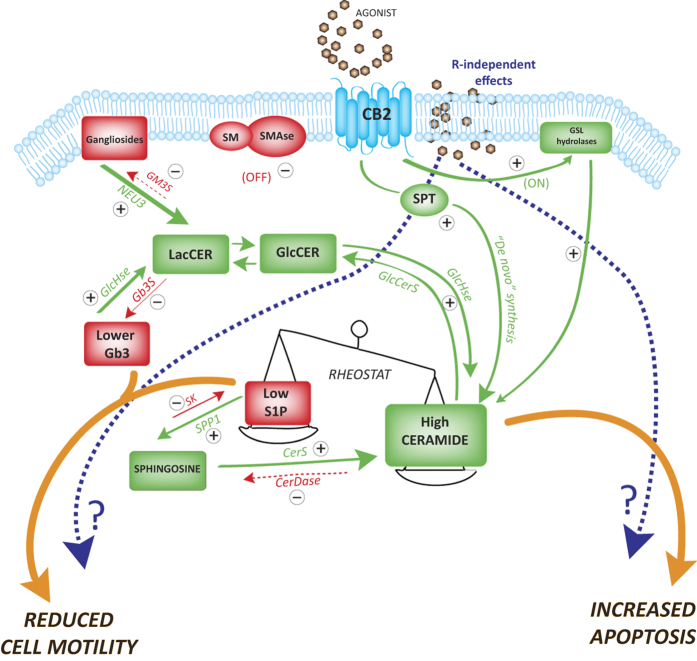
Molecular model of putative CB2-SL interaction in BC cells. Schematic representation of the molecular pathways involving CB2-mediated SL modulation on both apoptosis and cell motility of bladder cancer cells. Green and red arrows identify “on” and “off” pathways, respectively. SM: sphingomyelin; SMase: sphingomyelinase; SPT: serine-palmitoyl transferase; GlcCer: glucosylceramide; GlcCerS: glucosylceramide synthase; LacCERer: lactosylceramide; GM3S: GM3 synthase; Gb3S: Gb3 (globotriaosylceramide) synthase; S1P: sphingosine 1-phosphate; SK: sphingosine kinase; SPP1: S1P phosphatase; CERDase: ceramidase; CerS: ceramide synthase; GlcAse: glycohydrolases. Dashed arrows mean putative effects.

A putative molecular mechanism (Fig. 5) may imply that, upon CB2 activation, BC cells inhibit sphingosine kinase and upregulate ceramidase. This, in turn, favours a reduction of S1P and an increase of Cer that shift the cellular rheostat towards apoptosis. Concomitantly, the enzymatic hydrolysis of plasma membrane SL is activated and produces a signalling cascade that may modulate both cytoskeletal changes via p-ERM and cell migration via Src.

Limitations of the present study encompass the high variability and the low numerousness of the CB2 gene expression in human fresh specimens, which however show a significant difference when paired-analysed with relative healthy counterpart. Additionally, the receptor-independent effects of CB2 agonist^24^ and the role of GPR55 receptor were not comprehensively investigated.

In conclusion, our data associate the well-known anti-metastatic properties of cannabinoids^19^,^44^,^45^, with a molecular mechanism involving CB2 that induces SL metabolism changes. This finding and the selective tumour cytotoxicity may warrant further studies to support the use of non psycho-tropic CB2 agonists as novel adjuvant treatment in transitional BC, aimed at preventing both tumour recurrence and progression.

## Methods

### Ethical Statements

All the studies carried out on patient’s specimens were approved by the Institutional Ethical Committee (Ospedale San Raffaele, Milan, protocol URBBAN, Rev. February 2nd, 2014, approval date March 3rd, 2014) and the specific informed consent was obtained pre-emptively. We also declare that all the experiments involving animals were approved by the Institutional Animal Care and Use Committee (Ospedale San Raffaele, Milan, IACUC n. 631) and the Italian Ministry of Health (Office for Animal Health and Veterinarian Drugs). All the experimental procedures involving human or animal biologic material were carried out in compliance with the approved guidelines.

### Reagents and drugs

RPMI 1640 medium, trypsin-EDTA solution, MTT, protease/phosphatase inhibitor cocktail, and JWH015, were purchased from Sigma (Milan, IT). The fetal calf serum was from Gibco (Euroclone, Milan, IT). Primary antibodies anti-CB1, anti-CB2 and anti-FAAH, anandamide, rimonabant, SR144528, oleoyl ethyl amide and Annexin V/PI detection kit were from Cayman Chemical (Space Import, Milan, IT). CB2 gene silencing was carried out by using TriFECTa™ Dicer-Substrate RNAi kit (ID HSC.RNAi.N001841.12) according to manufacturer’s (Integrated DNA Technologies) directions. The primary antibody anti-GAPDH and HRP conjugated secondary antibodies were from Santa Cruz Biotechnology. Primary antibodies anti-cleaved Caspase-3, Ezrin EZR, pERM were from Cell Signaling Technology (Danvers, MA, USA), while anti SRC/pSRC were from AbCam. [1-^3^H]sphingosine and [^3^H]lipids used as chromatographic standards were kindly provided by Prof. Sonnino of the University of Milan.

### Immunohistochemical Analysis of Human bladder cancer

Archival material was obtained from internal pathologists. Histology was carried out on surgical specimens of 13 consecutive patients, not subjected to neoadjuvant chemotherapy, with transitional, muscle-invasive BC, which were fixed in 4% paraformaldehyde solution in PBS, paraffin embedded, cut at 4 μm and mounted on slides. As validation cohort, additional 40 cases were obtained from commercially available tissue micro arrays of bladder cancer with matched adjacent normal counterpart (US Biomax, Inc. Rockville, MD, USA). Sections were stained with either haematoxylin-eosin or for immunohistochemistry by using a LSAB+HRP Kit (DAKO). Immunohistochemical stainings were digitally scanned and analysed by 2 pathologists by using the Aperio system with colour deconvolution algorithm.

### Sample dimension, power calculation and statistics

A sample size of 19 cases was calculated from an expected effect size “d” of 0.7 (expression levels of normal vs tumour or CB1 vs CB2) tested with a paired 2-tailed T-test with a power of 0.80 and an α probability of 0.05 (G*Power Software). Statistical analysis was performed using GraphPad Prism Software, (San Diego, USA).

### Cell cultures

Human transitional carcinoma cell lines RT112 and RT4 were originally purchased from ATCC and further authenticated. Both tumour cell lines were maintained in RPMI 1640 medium supplemented with 10% FCS. Cells were routinely sub cultured by 0.05% trypsinization. Normal human fibroblast were kindly provided by Prof. S. Sonnino (University of Milan) and cultured as previously reported^46^. For cell viability assay, cells were seeded in quadruplicates in 96-well plates and incubated in 200 μl of complete RPMI to achieve 60% to 90% confluence. Cannabinoid agonists and/or antagonists were serially diluted (0.1–20 μM) into culture medium; cells were exposed to the compounds ranging from 24 to 120 hours. Cell viability was assayed by standard MTT incorporation. To perform metabolic labelling, cells were pulsed with 3 × 10^−8^ M [1-^3^H]sphingosine 24 hours after seeding in a culture dish. Two hours after, cells were replaced with fresh medium without [1-^3^H]sphingosine containing 20 μM JWH015 and further incubated for 72 hours. Under these conditions, all sphingolipids (including ceramide, sphingomyelin, neutral glycosphingolipids, and gangliosides) were metabolically radiolabelled at the steady state^47^.

### Immunoblotting analysis of protein patterns

Cells were lysed in ice-cold RIPA buffer containing protease and phosphatase inhibitors. Equivalent protein amounts were separated on SDS-PAGE gels and transferred onto PVDF membrane by elettroblotting. After blocking with 5% skim milk in TBS-T buffer, membranes were incubated overnight at 4 °C with primary antibody. After washing, 1 hour incubation with appropriate HRP-labeled secondary antibodies was done. Immunoblot was revealed by using a chemoluminescence detection kit (Life Technologies, Milan IT). Densitometric analysis was performed with ImageJ (NIH software).

### Lipid extraction and determination

Upon metabolic steady-state radiolabelling of cellular lipids with [1-^3^H]sphingosine, the content of radioactivity associated with each lipid was analysed. Radiolabelled cells were harvested in 2-ml ice-cold water and lyophilized. Cellular lipids were then extracted by using a chloroform/methanol/water solution (2:1:0.1 by vol). The total lipid extracts were subjected to a two-phase partitioning and processed as previously described^48^.

### Biochemical quantifications

Plasma membrane-associated enzymatic activities of β-galactosidase, β-glucosidase, GBA1 and, GBA2, β-hexosaminidase, and α-galactosidase were determined in living cells plated in 96-well microplates at a density of 3.3 × 10^4^ cells/cm^2^ by using specific fluorogenic substrates as previously described^49^. The artificial substrates were solubilized in DMEM-F12 medium (Invitrogen) without phenol red or serum at different final concentrations (see Suppl. Methods for details). At various times, aliquots of the medium were analyzed fluorometrically by using a microplate reader (Victor, Perkin Elmer) with the addition of 20 volumes of 0.25 M glycine (Sigma), pH 10.7. The data were expressed as n/pmoles of converted substrate/mg cells proteins/hr. Control experiments were performed to exclude any activity released from lysosomes and/or from other intracellular sites. The enzymatic activities associated with total cell lysates were determined by using fluorogenic substrates as previously described^17^. Sphingosine kinase 1 (SK1) activity was assayed as previously described^50^. For the analysis of S1P production, cells were pulsed with [1-^3^H]sphingosine for 45 min as previously described^51^.

### Gene expression analysis

Total RNA was isolated from the BC cells or snap-frozen tumour/normal adjacent tissue specimens by using a TRIzol Reagent kit (Invitrogen, Carlsbad, CA). Complementary DNA (cDNA) was synthesized from total RNA by using Super ScriptTM III Reverse Transcriptase (RT; Invitrogen). Thirty-five cycles were performed at 94 °C for 30 sec, 57 °C for 1 min and 72 °C for 1 min (see Suppl. Methods for primer sequences). The PCR products were separated on 1.5% (w/v) agarose gel and visualized by ethidium bromide staining. One μg of total RNA was reverse-transcribed into cDNA using a High Capacity cDNA kit (Applied Biosystems) according to the manufacturer’s protocol. Sybr-green RT-PCR was performed on an ABI Prism 7000 system (Applied Biosystems, Foster City, CA, USA) and the cycling conditions used were 95 °C for 15 sec, 60 °C for 1 min, for 40 cycles, followed by a melting point determination for dissociation curves. The relative amount of mRNA was calculated by using standard curves after normalization to the endogenous housekeeping genes GAPDH and HPRT1. PCR primers were designed with the Primer3 program to generate amplicons of 100–150 bp (see Suppl. Methods for sequences). The in silico analysis of CB2 was carried out by evaluating the TCGA data sets on bladder urothelial carcinoma (Provisional, 413 samples; Nature 2014, 131 samples) available on cBioPortal (http://bit.ly/2fDatNU).

### Wound-healing/invasion assays

Cells were plated at 90% confluence in 5%-serum DMEM. Twenty-four hours after seeding, the monolayers were scratched with a sterile plastic 200-μL micropipette tip, washed, and fed with culture medium containing the appropriate concentrations of DMSO or JWH015 compound. Cell-free area was quantified using ImageJ software. Three measurements were performed for each wound before the addition of agonists and, in a blinded fashion, after 24–48 hours of stimulation. Each experiment was performed in triplicate at least 3 times. Invasion assay was carried out in BioCoat Matrigel Invasion Chambers (BD Biosciences, Milan, Italy) according to the manufacturer’s protocol.

## Additional Information

**How to cite this article:** Bettiga, A. *et al*. Bladder cancer cell growth and motility implicate cannabinoid 2 receptor-mediated modifications of sphingolipids metabolism. *Sci. Rep.* **7**, 42157; doi: 10.1038/srep42157 (2017).

**Publisher's note:** Springer Nature remains neutral with regard to jurisdictional claims in published maps and institutional affiliations.

## Supplementary Material

Supplementary Information

## Figures and Tables

**Table 1:** Hydrolase activities associated with cell lysate and plasma membrane of RT4 and RT112 bladder cancer cells.

		Total (nmoles mg^−1 ^h^−1^)						Plasma Membrane-associated (pmoles mg^−1 ^h^−1^)				
		GBA1	GBA2	β-Hex	β-Gal	Neu3 §	SMase §	GBA1	GBA2	β-Hex	β-Gal	SMase
**RT4**	*BASAL*	3.2 ± 0.2	2.3 ± 0.1	2500 ± 157	186 ± 13	3.77 ± 0.4	250 ± 10	0.098 ± 0.001	0.0531 ± 0.009	0.569 ± 0.002	0.023 ± 0.002	2.8 ± 0.2
	* + JWH015*	4.8 ± 0.3	2.2 ± 0.1	1723 ± 145	166 ± 23	4.23 ± 0.32	153 ± 20	0.17 ± 0.01*	1,25 ± 0.03*	2.2 ± 0.2*	0.049 ± 0.001*	2.1 ± 0.3
**RT112**	*BASAL*	1.5 ± 0.2	1.1 ± 0.09	1150 ± 213	115 ± 8	8.23 ± 0.56°	103 ± 9	0.156 ± 0.001	ND	0.256 ± 0.003	0.0125 ± 0.001	2.5 ± 0.3
	* + JWH015*	1.7 ± 0.4	1.0 ± 0.07	650 ± 33	94 ± 8	10.6 ± 0.21*	123 ± 5	0.32 ± 0.03*	0.75 ± 0.02*	1.26 ± 0.01*	0.046 ± 0.003*	1.25 ± 0.05*

Hydrolases activities measured in both total cell lysate and cell PM of RT4 and RT112 cells treated or untreated with JWH015. Cells were treated or untreated for 48 hours with 20 μM JWH015 and subjected to enzymatic assays. The activity associated with the total cell lysate was evaluated as described above. The activity associated with the cell surface was measured directly on living cells. Data are expressed as pmol/nmol of formed metabolite/mg cell protein/hour and are the mean ± sd of at least three separate experiments. Comparisons between groups were made using Student’s t test. °p < 0.005 vs RT4 *p < 0.005 vs untreated cells. §: pmol mg^−1 ^h^−1^.
