# Supplementary material for: Bladder cancer cell growth and motility implicate cannabinoid 2 receptor-mediated modifications of sphingolipids metabolism
Source: Sci Rep. 2017 Feb 13;7:42157. doi: 10.1038/srep42157 (PMC5304189; doi:10.1038/srep42157)
Supplement: Supplementary Information [file srep42157-s1.pdf]

Bladder cancer cell growth and motility implicate cannabinoid 2 receptor-mediated modifications of sphingolipids metabolism

Arianna Bettiga[1#], Massimo Aureli[2#], Giorgia Colciago[1], Valentina Murdica[2], Marco Moschini[1], Roberta Lucianò[1,3], Daniel Canals[6], Yusuf Hannun[6], Petter Hedlund[1,4], Giovanni Lavorgna[1], Renzo Colombo[1], Rosaria Bassi[2], Maura Samarani[2], Francesco Montorsi[1,5], Andrea Salonia[1] and Fabio Benigni[1*].

SUPPLEMENTAL METHODS

rtPCR

The following primer pairs were used: hCB2 forward 5'- TATGGGCATGTTCTCTGGAA-3', reverse 5'-GAGGAGCACAGCCAACACTA-3'; hCB1 forward 5'-CGCTTTCCGGAGCATGTT-3', reverse 5'-TCCCCCATGCTGTTATCCA-3'; β-actin forward 5'-CGACAGGATGCAGAAGGAG-3', reverse 5'-ACATCTGCTGGAAGGTGGA-3'.

Real Time qPCR

The following primer pairs were used: GAPDH forward 5'-GGACTCATGACCACAGTCCA-3'; reverse 5'- CCAGTAGAGGCAGGGATGAT-3'; HPRT1, forward 5'- TGCAGACTTTGCTTTCCTTG-3'; reverse 5'-CTGGCTTATATCCAACACTTCG-3'; CB1, forward 5'- CCAGCAGACCAGGTGAACAT-3'; reverse 5'-GGGTTCAGGACCATGAAACACT-3'; CB2 , forward 5'-CCTGTTCATTGGCAGCTTGG-3'; reverse 5'-TCAGCAGGAAGACAGCCTTG-3'; alpha 1,4-galactosyltransferase (A4GALT), forward 5'-TCTGCACCCTGTTCATCATC-3'; reverse 5'- CTTTCTCCTTGGGCTCTCC-3'; ceramide synthase 2 (CERS2), forward 5'- TCCGACTGGGACTCTTAATC; reverse 5'- TGTTGCAGGTGTTCTTCCAT-3'; ceramide synthase 3 (CERS3), forward 5'-GAATCAAGAGAGGCCTTCCA-3'; reverse 5'-GCAATTCCAGCAACAGTGAT; membrane sialidase 3 (NEU3), forward 5'-CCTGAAGCCACTGATGGAA-3'; reverse 5'-TTCCTGCCTGACACAATCTG-3'; Lactosylceramide alpha-2,3-sialyltransferase (ST3GAL5); forward 5'-AATTGGCGCTGTTATTTGAGC-3'; reverse 5'-CTGGCAAGAGTTCCAAGAGG-3'; sphingosine kinase 1 (SPHK1), forward 5'-CCCCAGCAAACCGGACCGAC-3'; reverse 5'-CCCCAGCAAACCGGACCGAC-3'; sphingosine kinase 2 (SPHK2), forward 5'-CCCCTCAGACTCAGCGGCCT-3'; reverse 5'-GTGGGCGAGGCAGGTTCCAC-3'.;

Orthotopic bladder cancer model

The orthotopic animal model was performed as follows: Luciferase-expressing RT112 (RT112-luc) cells were generated by using ready-to-use lentiviral particles (Redifect, Perkin Elmer). Immunodeficient nude mice were then anesthetized, laparatomized to expose the bladder, and injected transmurally with RT112-luc cells ($2.5x10^5$ cells in PBS/matrigel 1:2, 50 μL)Three days post tumor implantation, 15 min after luciferase substrate administration, animals underwent to *in vivo* imaging for assessing tumor take and started intravesical instillation of therapy. Drug (JWH015,20 μM) or vehicle (PBS) instillations were repeated at day 7, and 11 and 15 post tumor injection. Mice were carefully monitored daily for health status and hematuria and tumor burden was observed by luminescence scan at day 10, 28, and 48 post tumor implantation. Animals were terminally euthanized to allow bladder histopathological evaluation.

Biochemical quantifications

Artificial substrates concentrations: 0.25 mM for 4-methylumbelliferil-β-D-galactopyranoside (MUB-Gal), 6 mM for 4-methylumbelliferil-β-D-glucopyranoside (MUB-Glc), 1 mM for 4-methylumbelliferil-N-acetyl-β-D-glucosaminide-6-sulfate (MUGS) and 4-methylumbelliferil-α-D-galactopyranoside (MUB-α-Gal) (Glycosynth, Warrington, UK). DMEM-F12 was adjusted to pH 6 for the detection of β-galactosidase, conduritol-β-epoxide (CBE)--sensitive β-glucosidase (GBA1), and β-hexosaminidase, and to pH 6.2 for the detection of β-glucosidase, GBA2 and α-galactosidase. For CBE-sensitive β-glucosidase and GBA2 assays, the cells were pre-incubated for 30 minutes at room temperature with 5 nM AMP-dNM (adamantane-pentyl-dNM); N-(5-adamantane-1-yl-methoxy-pentyl)-deoxynojirimycin) (Overkleeft et al 1998) andor 1 mM CBE (Sigma) in DMEM-F12, respectively, and then incubated at 37°C with gentle shaking.

SUPPLEMENTAL RESULTS:

1. Validation of CB1 and CB2 antibodies.

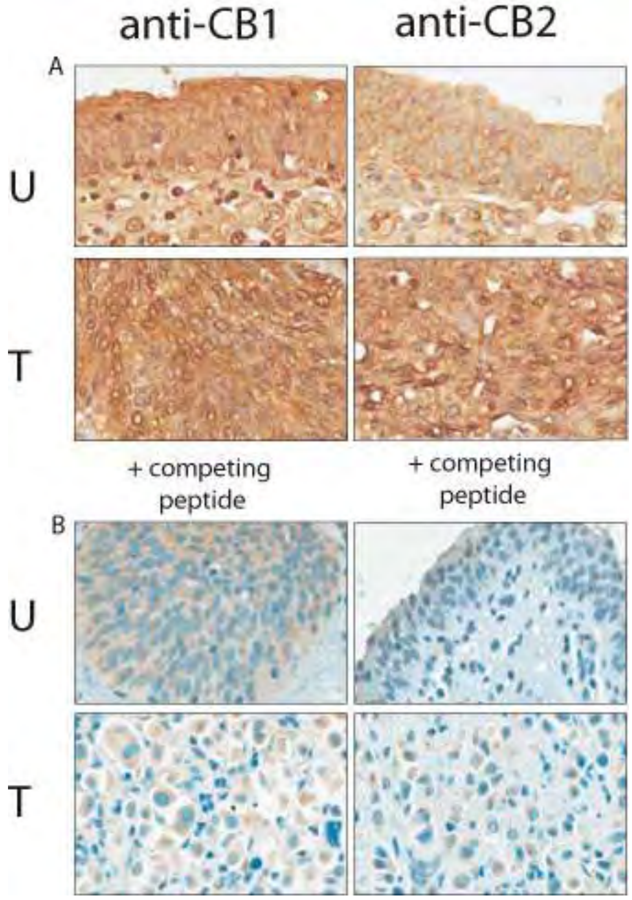

***Suppl. Fig.s1. Competing cannabinoid receptor staining**.*

Immunohistochemical analysis of CB1 and CB2 receptor expression in human normal urothelium (U) and muscle invasive urothelial tumor (T), on the same section (magnification 200X). Panel B: Controls with Ab-specific blocking peptides. The source of samples is described in Methods.

2. Biochemical characterization of BC cellular models

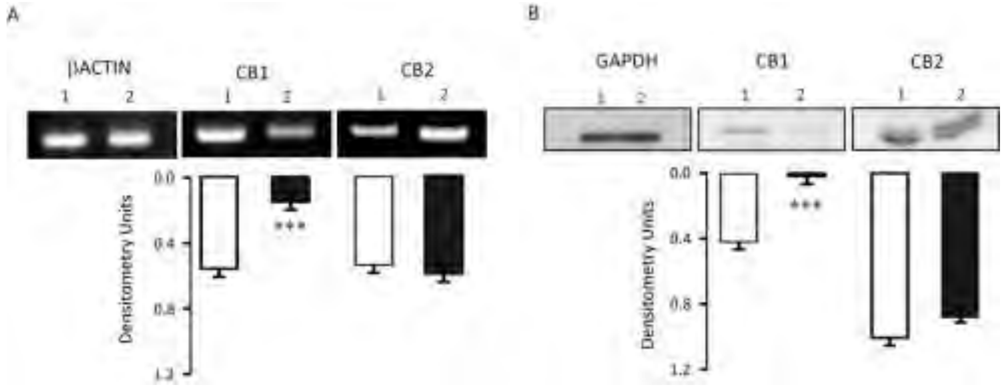

Suppl. Fig.s2. Expression of CB in BC cell lines.

*Expression of cannabinoid receptors assessed by RT-PCR (A) and Western blot (B) in RT4 (lane 1) and RT112 (lane 2) cells. The densitometric analysis of the relative bands for CB1 (66 bp or 60kDa) and CB2 (141 bp or 40-45 kDa), compared to reference housekeeping gene (βactin, 137 bp or GAPDH, 39 kDa), from three separated experiments is shown (*** p<0.001 vs lane 1, Student's T test).*

3. Genomic and bioinformatics analysis of CB2 in urothelial bladder cancer.

Genetic profiling of CB2 (CNR2) was carried out on the TCGA data sets encompassing either 413 cases (http://bit.ly/2fDFqSi) or 131 samples (http://bit.ly/2fZzrvs) of urothelial bladder cancer by using the cBioPortal analysis tools[1]. The CNR2 gene was found to be mutated in 3 patients (0.7%), whereas an alteration of the CB2 copy number (gain, amplification, and shallow deletion) was present in 154 samples (35.6%), with the majority of them being T3-T4 stage. No clear correlation was found between CNR2 expression levels and either survival or diagnosis subtype (papillary or non-papillary). Finally, by evaluating the expression of CNR2 gene within the recently identified mRNA cluster subtypes in BC [2], in all the cases available in the public domain, we found that higher CNR2 mRNA expression significantly associated with cluster mRNA subtype IV (49.1±30 expr. units, n=13, $p<0.05$ 1-way ANOVA with Tuckey post-test vs cluster 1: 0.81±0.4, 2: 4.6±1.8, and 3: 1.6±0.7). Additionally, we found weak but significant positive correlation between CNR2 gene expression and miR-100-5p (Pearson 0.22, p=0.03) and an anti-correlation with E-Cadherin protein expression (Pearson -0.25, p=0.01), both features of type IV cluster. Further cases on mRNA clustering are not presently available, as confirmed by personal communication from TCGA group.

4. Relative contribution of CB1 and CB2 to agonist-induced cytotoxicity

A dual CB1-CB2 agonist (anandamide, AEA) was used. As expected, AEA (20 µM) displayed a cytotoxic effect that peaked 48 hrs after exposure. Blockade of CBs with rimonabant (CB1 antagonist) or SR144528 (CB2 antagonist) in AEA-treated RT112 cells suggested that both receptors contributed to the early CB agonist-induced cytotoxic effects, since both CB1 and CB2 antagonists partially rescued cell viability to 50% 24 hrs after AEA exposure. Only CB2 receptor blockade, however, prevented cell death at longer time points, with a 75% increase of cell viability measured 48 hrs after AEA. Cells exposed to AEA spontaneously regained normal proliferation activity after 5 days (data not shown). Moreover, when cells were co-treated with AEA plus oleoyl ethyl amide (OEtA), a specific blocker of FAAH enzyme, the cytotoxic effect of AEA was potentiated (data not shown), suggesting that the reduced effects of AEA measured 5 days after exposure were mainly due to its degradation.

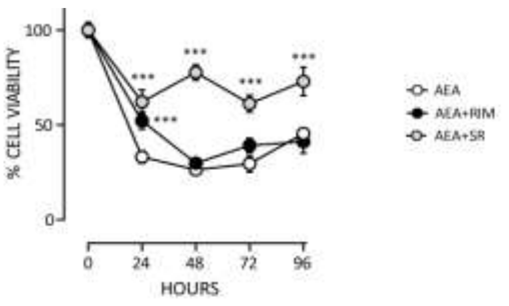

Suppl. Fig. s3: Role of CB1 and CB2 in cytotoxicity.

*Antagonistic effect of CB1-specific rimonabant (RIM, 10 µM) and CB2-specific SR144528 (SR, 10 µM) drugs given simultaneously with AEA (20 µM) on CB1/CB2 agonist- induced toxicity of RT112 cells. Concentration of agonist and antagonists were chosen after preliminary experiments to determine optimal agonist cytotoxic effect and reliably measurable blocking effect of the antagonists when simultaneously co-administered to cells. *** p<0.001 vs AEA alone, 2-ways ANOVA, Bonferroni posttest.*

5. <u>In vivo effect of CB2 agonist instilled othotopically in xenotrasplanted immunodeficient mice</u>

The antitumor effect of cannabinoid agonist JWH015 was tested *in vivo* in mice xenotrasplanted with RT112-luc cells, implanted intramurally. Four intravesical instillation of JWH015 (20 μM), carried out 4 days apart, significantly reduced the tumor burden 24 and 48 days after tumor implantation. *JWH015 treatment, however, did not completely eliminate the tumor, which 33 days after last intravesical instillation, displayed re-growth (Fig. s4A). The histological image of the bladder (Fig. s4C) shows a proliferating neoplastic focus within the muscle layer thickness.*

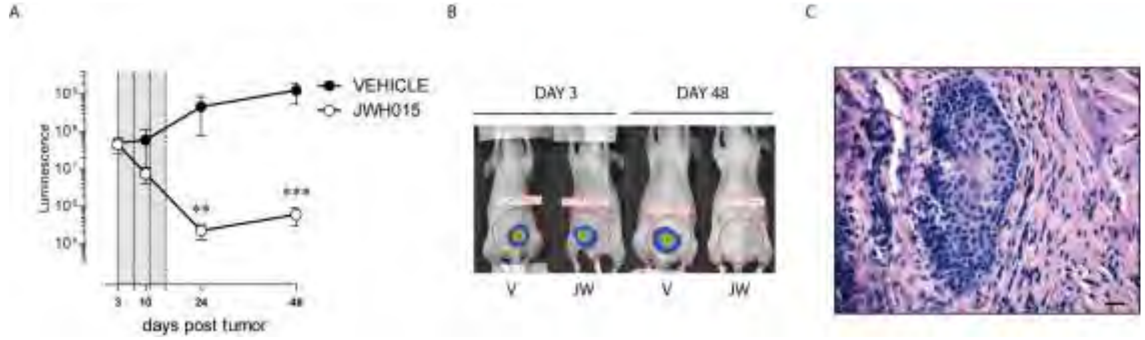

Suppl. Fig. s4. Effect of JWH015 on orthotopic bladder tumor growth in vivo*.*

*Panel A: Quantification of tumor-associated luminescence over time after intramural injection of RT112-luc cells. Vertical dashed lines indicate intravesical treatment (day 3, 7, 11, and 15). Data are represented as mean±SE of individual animal bioluminescence (n=8); ** p<0.01, *** p<0.001 vs vehicle, two-way ANOVA with Bonferroni multiple comparison post test. Panel B: Imaging of representative animals taken 3 and 48 days after tumor implantation and vehicle (V) or JWH015 (JW) treatment. Panel C: Terminal histological analysis (haematoxylin/eosin stain) of the bladder from representative JWH015-treated animal. Scale bar= 25 μm.*

6. <u>The apoptotic phenotype of BC cells after CB2 agonist exposure</u>

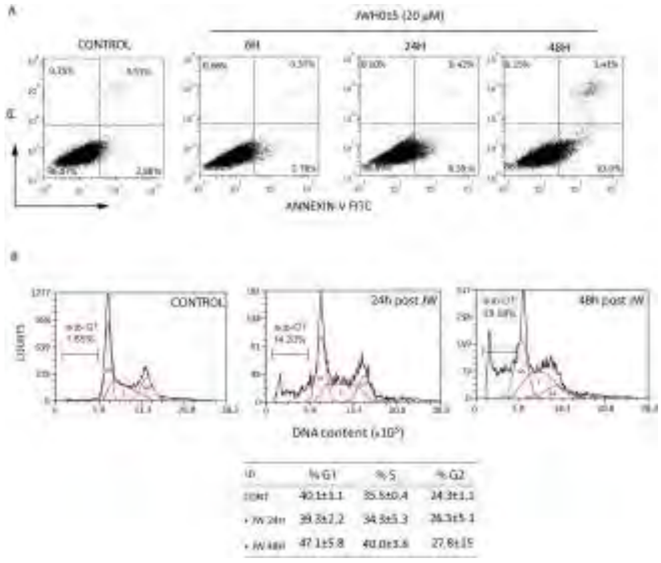

Suppl. Fig. 5. CB2 agonist-induced apoptosis.

Representative FACS plots of annexin V/PI + cells induced by JWH015 exposure over time (Panel A). Panel B: cell cycle analysis of RT112 cells treated with JWH015 (20 μM). The frequency of sub G1 phase population is shown. The table summarizes the % of cells for each phase. Histogram plots are overlaid with the interpolation model used to calculate the cell cycle phases (red line).

7. Defective autophagy induced by CB2 agonist.

Cannabinoids can promote cell death in tumours by inducing autophagy and modulating membrane sphingolipids [3,4]. JWH015 induced conversion from the soluble form of LC3 (LC3-I) to autophagosome-associated form (LC3-II) and mediated inhibition of mTORc1, evaluated as reduced phosphorylation of p70S6, was detected already 6 hrs after agonist stimulation and counteracted by the autophagosome fusion inhibitor bafilomycin. The data suggest that some molecular events, such as mTORC1 inhibition, predisposing the autophagy machinery, are occurring before the cannabinoid-mediated caspase 3 activation, confirming that cellular and possibly defective autophagy, is preceding the JWH015-induced cytotoxicity of BC cells.

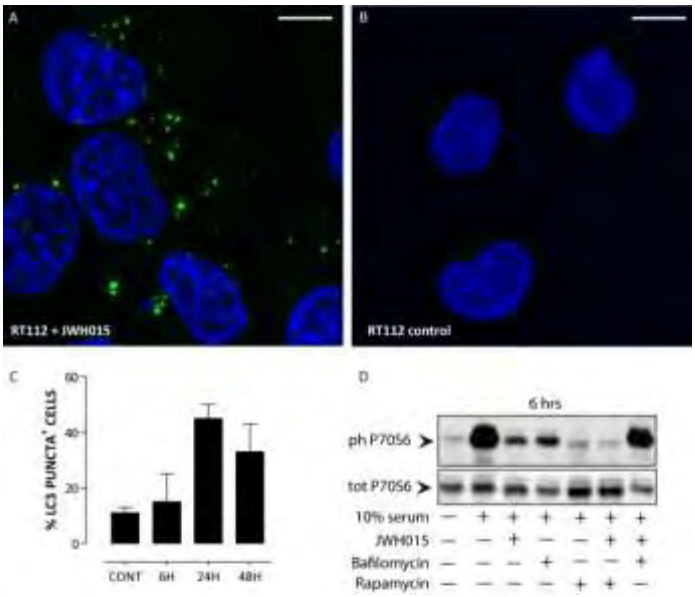

Suppl. Fig. s6. Autophagy occurring in RT112 upon exposure to CB2 agonist.

The conversion of the soluble form of LC3 (LC3-I) to the lipidated and autophagosome-associated form (LC3-II) was evaluated on RT112 cells upon 24 hrs exposure with JWH015 (20 µM) (Panel A) in comparison to the vehicle-treated control (Panel B). Fluorescence microscopy analysis was carried out by using a specific LC3 antibody followed by alexa-green labeled secondary pAb. Nuclei were labeled with DAPI (blue). Scale bars= 4 µm. The insoluble form of LC3 is evidenced by more intensely and condensed green-labeled intracellular localizations. Quantification of LC3 puncta^{+} cells is shown in Panel C. The percentage of cells with more than 5 LC3 punctae was calculated by counting at least 6 fields. Data are the mean±sd of quadruplicate counts in two independent experiments. Panel D: immunoblot analysis of p70SP phosphorylation in RT112 cells measured 6 hrs after JWH015 challenge (20 µM) with or without 30 min pre-treatment with rapamycin (20 nM) or bafilomycin (100 nM).

8. Sphingolipids composition of RT4 and RT112 cells before and after CB2 agonist challenge

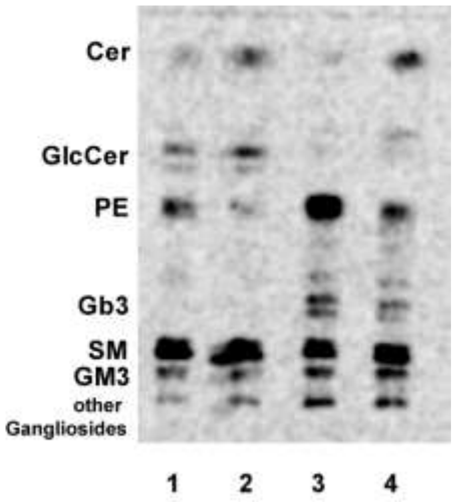

Suppl. Fig. s7. Representative TLC showing lipid composition of BCa cells before and after JWH015 treatment*.*

After metabolic labeling with 2h pulse of [1-3H]sphingosine, RT4 (lanes 1–2) and RT112 (lanes 3-4) cells were chased with vehicle (lanes 1 and 3) or 20µM JWH015 for 24 h (lanes 2 and 4). Radioactive lipids were identified by using the appropriate standards: ceramide (Cer), glucosylceramide (GlcCer), phosphoethanolamine (PE), globotriaosylceramide (Gb3), sphingomyelin (SM)

9. Functional phenotype of low- (RT4) and high- (RT112) aggressive BC cell lines

As shown in suppl. Fig. S7, the levels of the globotriesosylceramide, Gb3, a cell motility-promoting sphingolipid, were 15-fold higher ($p<0.001$) in RT112 than in RT4 cells. Conversely, RT4 cells showed 7-fold higher levels ($p< 0.01$) of glucosylceramide (GlcCer). We also detected increased amounts of gangliosides typically associated with reduced invasiveness, particularly GM2 and GM3, in RT4 cells. The SL profile was also evaluated in T24, HT1376, and 5637 BC cell lines and found similar to RT112 (not shown). The different SL pattern between RT112 and RT4 cell lines were, in turn, associated to different cellular motility, which was independently of their comparable proliferation rates (not shown). In a wound-healing assay, RT112 cells reconstituted more than 70% of the scratched area within 48 hrs (suppl. Fig.8 B-C), while RT4 cells only populated 5%. The metastatic features of RT112 cells were also markedly different from the RT4 cells, as demonstrated by a classical matrigel-based invasion assay (suppl. Fig.8D). In addition to these evidences, along with the recent findings demonstrating the strong link between plasma membrane SL and the activation of ERM proteins in driving cancer progression mechanisms [27,28], we showed that the levels of phospho-ERM detected in RT112 cells were significantly higher than RT4 (suppl fig Fig.8E), suggesting a cytoskeletal rearrangement in high-grade BC cells. Previous data [5] showed that the phosphorylation level of ERM was strictly linked to the level of S1P. Consistently with this, the content of S1P we measured in high-motility RT112 cells was 3-fold higher than the one in low-motility RT4 cells (3.8 ± 0.56 vs 1.43 ± 0.36 nCi $mg^{-1;}$ $p<0.01$). Furthermore, the sphingosine kinase 1 (SK1) was upregulated in both gene expression (5-fold, not shown) and enzymatic activity (891 ± 94 vs 283 ± 71 pmoles $hour^{-1}$ $mg^{-1;}$ $p<0.01$) in RT112 cells compared to RT4 cells.

Finally, in order to directly relate the SL metabolism with cell motility, RT112 cells were pre-treated with an inhibitor of SL synthesis (AMP-dNM), before measuring the cell migration in the scratch assay. As shown in suppl. Fig.8F, AMP-dNM was able to significantly reduce the motility of RT112 cells.

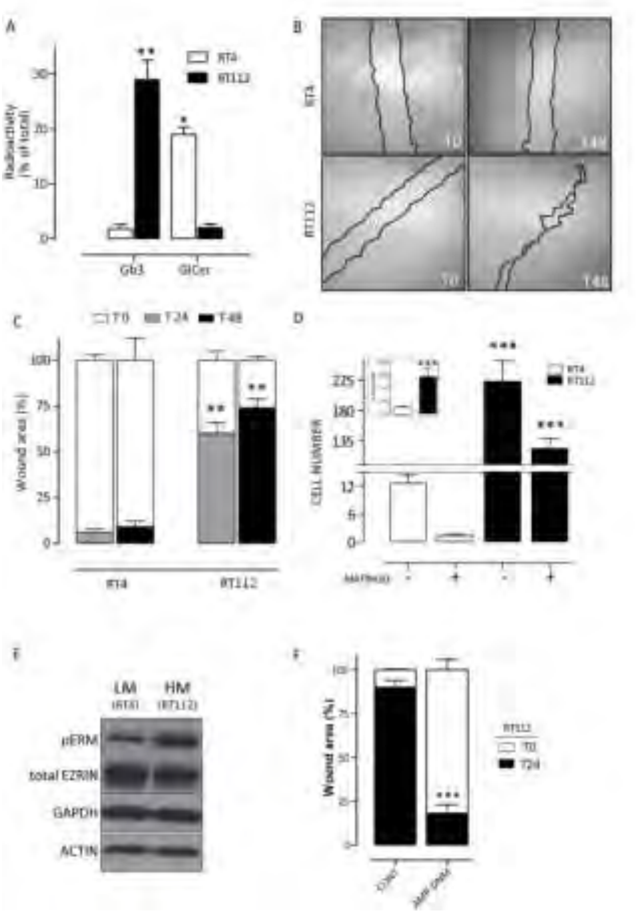

Suppl. Fig s8: Differential GSL composition and motility between high- (RT112) and low- (RT4) aggressive BCa cells.

*Panel A: Sphingolipid composition of RT4 and RT112 cells. Cell lipids were metabolically labeled with [1-3H]sphingosine at the steady-state. Lipids were then extracted separated by HPTLC, visualized by digital autoradiography and quantified with specific Beta-Vision software. Data are expressed as the percentage of total radioactivity incorporated into sphingolipids and are the means ± SD of three different experiments. * p< 0.05 versus RT112. ** p< 0.01 versus RT112. Panel B: Representative pictures of wound-healing assays in either RT4 cells or RT112 cells at time 0 (T0) and 48 h after scratch (T48). Wound edges were artificially highlighted by drawing a black line. Panel C: Quantification of cell migration evaluated as % of cell-covered area at 24 hours (grey bars) and 48 hours (black bars) compared to the cell-free wound area at time 0 (white bars). Data are the mean±sd of three independent experiments done in triplicate. ** p<0.01 vs RT4 cell, ANOVA, Dunnett's T test. Panel D: invasion assay on RT4 and RT112 cells. Data aree expressed as number of migrated cells through the pores with or without the presence of matrigel. The percent of invasion through the matrigel is show in the panel inset. Data are the mean±sd of quadruplicates from 2 independent experiments. *** p< 0.01 vs RT4 cells, Student' T test. Panel E: Phospho-ERM immunoblot quantification on low-motility (LM, RT4) and high-motility (HM, RT112) cells. EZRIN, GAPDH and β-actin proteins were used as control. Panel F: Cell motility quantification of RT112 cells in the presence of SL metabolism modulator evaluated as % of re-populated scratched area,*

compared to control (CONT). Wounded RT112 cultures were incubated with vehicle (DMSO) or AMP-DNM (0.4 µM) for 24 hrs. Data are the mean±sd of three experiments done in triplicate.

References

1 Gao, J. *et al.* Integrative analysis of complex cancer genomics and clinical profiles using the cBioPortal. *Sci Signal* **6**, pl1, doi:10.1126/scisignal.2004088 (2013).

2 Network, C. G. A. R. Comprehensive molecular characterization of urothelial bladder carcinoma. *Nature* **507**, 315-322, doi:nature12965 (2014).

3 Salazar, M. *et al.* Cannabinoid action induces autophagy-mediated cell death through stimulation of ER stress in human glioma cells. *J Clin Invest* **119**, 1359-1372 (2009).

4 Velasco, G. *et al.* Cannabinoids and ceramide: Two lipids acting hand-by-hand. *Life Sciences* **77**, 1723-1731, doi: 10.1016/j.lfs.2005.05.015 (2005).

5 Gandy, K. A. O. *et al.* Epidermal growth factor-induced cellular invasion requires sphingosine-1-phosphate/sphingosine-1-phosphate 2 receptor-mediated ezrin activation. *Faseb Journal* **27**, 3155-3166, doi:Doi 10.1096/Fj.13-228460 (2013).
